# Oh what a tangled web we weave when first we practice to deceive…

**DOI:** 10.1192/j.eurpsy.2021.89

**Published:** 2021-08-13

**Authors:** M. Wise

**Affiliations:** Psychiatry, Brent CMHT, London, United Kingdom

## Abstract

‘Oh what a tangled web we weave when first we practice to deceive’. Marmion, Sir Walter Scott 1808. Conflict is unpleasant, it is aversive, we tend to avoid it. Yet inevitably tension between individuals or between individuals and society is inevitable as the wants of one collide with the purpose of the other. Most of these tensions resolved peacefully but a societal level aggression can sometimes spill out. In the hinterlands between individuals and larger groups these can play out more safely through the courts or sometimes the avoidance of conflict can be the only tactic that the individual can use. As doctors we are used to sing medical problems with patients have true disease believe they have two disease and want to get well-the standard social model of medicine. But sometimes this plays out differently there are those who may fabricate symptoms to avoid punishment or for reward: malingering. There are those who believe they have a disease but the distress is disproportionate to any possible recognised component; somatic symptom disorder. There are those whose anxiety about whether they have a disease or not is paralysing and perhaps most distressing for all of the groups who self-harm or malinger with authentic illness or disease. In this talk Dr Wise will, using case examples, look at a couple of the tools that exist to assist psychiatrists in piloting a pathway through the stormy waters of abnormal illness in litigation.

**Disclosure:**

No significant relationships.

